# Novel IVS7+1G>T mutation of life-threatening congenital factor VII deficiency in neonates: A retrospective study in China

**DOI:** 10.1097/MD.0000000000017360

**Published:** 2019-10-04

**Authors:** Juan He, Wei Zhou, Hui Lv, Li Tao, XiaoWen Chen, Ling Wang

**Affiliations:** a The First Affiliated Hospital of Jinan University; b Department of Neonatology, Guangzhou Women and Children's Medical Center, Guangdong, China.

**Keywords:** congenital factor VII deficiency, genetic diagnosis, intracranial hemorrhage, neonatal

## Abstract

In neonates, congenital factor VII deficiency (FVIID) is characterized by central nervous system bleeding and gastrointestinal hemorrhage, often resulting in poor prognosis and high mortality.

To improve understanding of FVIID in neonates in Asia, we retrospectively analyzed the clinical manifestations, diagnosis, treatment, clinical course, and genetic diagnosis of 2 cases of neonatal FVIID in the Department of Neonatology, Guangzhou Women and Children's Medical Center, Guangzhou, China, from January 2007 to December 2017 and performed a review of the relevant literature.

Both neonates were female and presented with severe gastrointestinal tract and intracranial hemorrhage. The laboratory findings were characterized by repeated and non–vitamin K1-dependent prolonged of the prothrombin time (PT), Factor VII (FVII) activity was 1.5% and 3%, respectively. Both neonates died of severe intracranial hemorrhage, at 31 days and 6 months after birth, respectively. Gene sequencing results revealed a homozygous mutation in the FVII gene splice site (IVS7+1G>T) in both cases. Upon review of relevant literature published since 1996, we identified 19 cases of neonatal FVIID. The patients were full-term neonates with onset of symptoms mostly within 7 days after birth (73.7%), which included gastrointestinal bleeding (blood stool, vomiting blood; 31.6%), nervous system signs (drowsiness, convulsions, poor response; 26.3%), severe intracranial hemorrhage (84.2%), significantly prolonged PT with significantly decreased FVII activity (89.5%), high mortality, and disability (68.4%). Gene sequencing was performed in 9 of the 19 children evaluated and revealed a mutation in the FVII gene in all cases.

FVIID can be clinically diagnosed based on the presence of prolonged PT that is difficult to correct and significantly decreased FVII activity (≤5%). As mutations in some sites are associated with severe bleeding, genetic diagnosis represents a useful tool for prenatal diagnosis of FVIID. In brief, we should pay great attention to the FVIID onset of the neonatal period, although it is rare but result in life-threatening bleeding with poor prognosis.

## 1 Introduction

Congenital factor VII deficiency (FVIID) (F7:OMIM:217500) is an autosomal recessive hereditary disorder caused by functional defects in factor VII (FVII) that affect the initial stage of the extrinsic coagulation pathway, resulting in organ bleeding.^[1]^ The incidence of approximately 1/500000, 18% of patients have a family history of consanguineous marriage.^[2]^ Adult FVIID presented with minor or post-traumatic hemorrhages such as epistaxis, skin and mucosal petechiae, gingival bleeding, menorrhagia, and persistent bleeding after trauma.

In the neonatal period, the onset of FVIID is characterized by central nervous system bleeding and gastrointestinal hemorrhage.^[3,4]^ FVIID is often accompanied by serious neurological complications, resulting in poor prognosis and high mortality.^[5]^ There is no radical treatment for FVIID currently, therapeutic strategies mainly rely on infusion of fresh frozen plasma, prothrombin complex concentrates, and human recombinant activating FVII, but the outcomes have been inconsistent.^[6]^ We retrospectively analyzed 2 cases of FVIID managed at the Guangzhou Women and Children's Medical Center and confirmed by genetic diagnosis. Additionally, we reviewed relevant literature and summarized the clinical characteristics, diagnosis, treatment, and clinical course of FVIID. We hope that our report raises awareness of this rare but life-threatening condition.

## 2 Case presentation

### 2.1 Case 1

The first patient was a female neonate aged 15 days, who was the second child of her mother and had been born at the gestational age of 37 weeks. The patient was admitted to our hospital because of repeated bloody stool for 1 week and fever for 1 day. On the 7th day after birth, the child presented with bloody stool and mild hematemesis. On the 9th day, she was hospitalized at a local hospital. Blood tests revealed anemia, significantly prolonged prothrombin time (PT), and normal activated partial prothrombin time (APTT). The symptoms improved with symptomatic treatments such as vitamin K1 supplementation, but the child developed fever on the 14th day after birth, with a peak body temperature of 38.4°C, but without convulsion. Magnetic resonance imaging (MRI) of the head revealed a small amount of subarachnoid hemorrhage. The parents and brother were in good health, and the parents denied any family history of hereditary diseases or bleeding disorders, as well as consanguineous marriage.

Upon admission at our hospital, physical examination revealed a body temperature of 38.1°C, breathing rate of 46 breaths/minute, heart rate of 148 beats/minute, and blood pressure of 89/48 mmHg. The child presented with skin pallor, scattered ecchymosis of the limbs, and softened anterior fontanelle. The bilateral pupils were equal in size and reactive to light. There were no abnormalities in the heart or chest, and no abdominal bulging. The liver and spleen were not palpable under the ribs, and bowel sounds appeared normal. Blood tests revealed leukocytes at 14.1 × 10^9^ cells/L, red blood cells at 3.20 × 10^12^ cells/L, hemoglobin at 97 g/L, and platelets at 458 × 10^9^ cells/L. Coagulation tests revealed PT as 40.80 seconds, APTT as 42.9 seconds, thrombin time as 16.4 seconds, fibrinogen level as 3.87 g/L, high-sensitivity C-reactive protein as 10.17 mg/L, and procalcitonin as 0.34 ng/mL. Liver function tests revealed total protein level as 53.1 g/L, albumin as 34.3 g/L, globulin as 18.8 g/L, total bilirubin as 40.8 μmol/L, and direct bilirubin as 15.9 μmol/L. There were no abnormalities in the results of urine tests, stool tests, blood gas electrolyte analysis, hemolytic test, or antibody tests for *Toxoplasma gondii*, rubella virus, cytomegalovirus, and herpes virus. Cranial MRI revealed a small amount of subarachnoid hemorrhage in the left frontal Jobes, as well as occipital horn hemorrhage in the bilateral ventricles.

**Figure 1:**
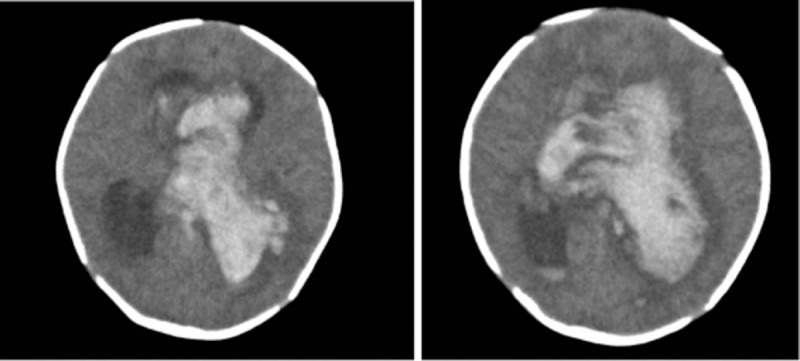
Head computed tomography scans of a female neonate admitted for bloody stool and fever (case 1). Hemorrhage is noted in the left temporal lobe,with invasion into the supratentorial ventricle, which appeared dilated and filled with blood (especially in the left ventricle).

The patient was started on symptomatic treatments including administration of vitamin K1, plasma, cryoprecipitate, and cefoperazone sulbactam, but PT did not improve (33.8–45.6 seconds). The activity of FVII was 1.5%, whereas the activities of other clotting factors were normal. The patient was thus diagnosed with FVIID and was started on treatment with prothrombin complex concentrates. After 4 days of treatment, PT had reduced to 26.9 s, and no enlarged hemorrhage site could be detected on MRI reexamination. The patient was discharged after 13 days of hospitalization. On the 3rd day after discharge, the patient became irritable and started rejecting milk, but did not exhibit convulsions. Upon presentation to the local hospital, skull B-ultrasound revealed significant dilatation of the ventricles and abnormal PT. The patient was later transferred to the emergency department of our hospital, presenting with convulsions and coma. Head computed tomography revealed hemorrhage in the left temporal lobe,with invasion into the supratentorial ventricle, which dilated and filled with blood (Fig. 1). The patient died despite cardiopulmonary resuscitation.

### 2.2 Case 2

The second patient was a female neonate aged 10 days, who was her mother's first child and had been born at the gestational age of 39 weeks. The patient was hospitalized for weak cry and poor response for 1 day. On the 3rd day after birth, the child was admitted to a local hospital for vomiting of coffee-ground-like material and bloody stool, but was discharged after 4 days of treatment including fasting and hemostatic therapy. On the 9th day after birth, the child exhibited weak cry and poor mental response and thus presented to our hospital for medical treatment. The parents were in good health and denied any family history of hereditary diseases or bleeding disorders, denied consanguineous marriage. Physical examination revealed a body temperature of 36.5°C, breathing rate of 28 breaths/minute, heart rate of 125 beats/minute, and blood pressure of 78/45 mmHg. The patient had poor response, weak cry, and severe jaundice. There were no bleeding spots or ecchymosis of the skin. The patient had full anterior fontanelle and irregular breathing, although no abnormalities in the heart or lung could be detected on auscultation. There was abdominal bulging and softening with no abdominal mass and with normal bowel sounds. Although the muscle tension of the limbs was normal, the rooting, sucking, and holding reflexes were weakened. Blood tests revealed a leukocyte count of 14.3 × 10^9^ cells/L, red blood cell count of 2.54 × 10^12^/L, hemoglobin level as 85 g/L, and platelet count of 774 × 10^9^ cells/L. Coagulation tests revealed PT as 40.40 seconds, APTT as 36.40 seconds, thrombin time as 16.7 s, fibrinogen level as 4.13 g/L, and high-sensitivity C-reactive protein level as 1.52 mg/L. Liver function tests revealed total bilirubin level as 319.6 μmol/L, direct bilirubin as 29.6 μmol/L, and indirect bilirubin as 290.0 μmol/L. Cranial MRI revealed multisite hemorrhage in the ventricles, bilateral cerebral subarachnoid, and posterior cranial fossa, as well as under the cerebellar tentorium.

**Figure 2:**
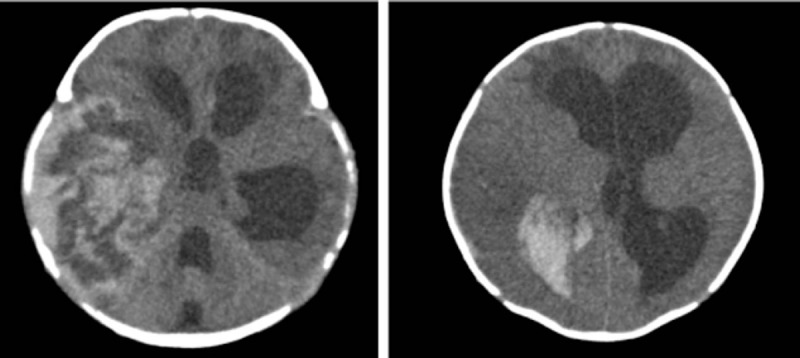
Head computed tomography scans of a female neonate hospitalized for weak cry and poor mental response (case 2). Multiple hematomas are visible in the right iliac occipital lobe and bilateral frontal lobes. The midline appears shifted slightly to the left. Hemorrhage is noted in the right lateral ventricle horns and body, with supratentorial hydrocephalus.

The patient was initiated on treatment with fasting, restrictive transfusion, dehydration, phototherapy, and administration of vitamin K1, red blood cells, plasma, and cryoprecipitates, but PT did not improve (29.7–41.1 s). Repeated apnea occurred, with poor response. The activity of FVII was 3%, whereas the activities of other clotting factors were normal. The patient was thus started on treatment with prothrombin complex concentrates. External ventricular drainage was recommended by the surgeons from the department of brain surgery. However, the parents decided to stop the treatment and the patient was discharged against medical advice. Two months after discharge, the patient was readmitted for convulsions and fever. Head computed tomography revealed multiple hematomas in the right temporal and parietal lobe and bilateral frontal lobes; the midline appeared shifted slightly to the left, with hemorrhage in the right lateral ventricle horns and body; supratentorial hydrocephalus was also noted (Fig. 2). After external ventricular drainage, cranial computed tomography revealed an improvement in bleeding, and the patient was discharged. The patient died at the age of 6 months, because of recurrent intracranial hemorrhage (Table 1).

**Table 1:**
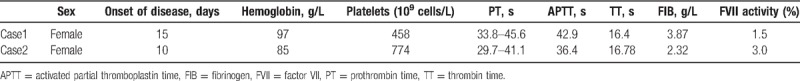
Clinical data of described 2 patients.

### 2.3 Genetic mutation analysis

**Figure 3:**
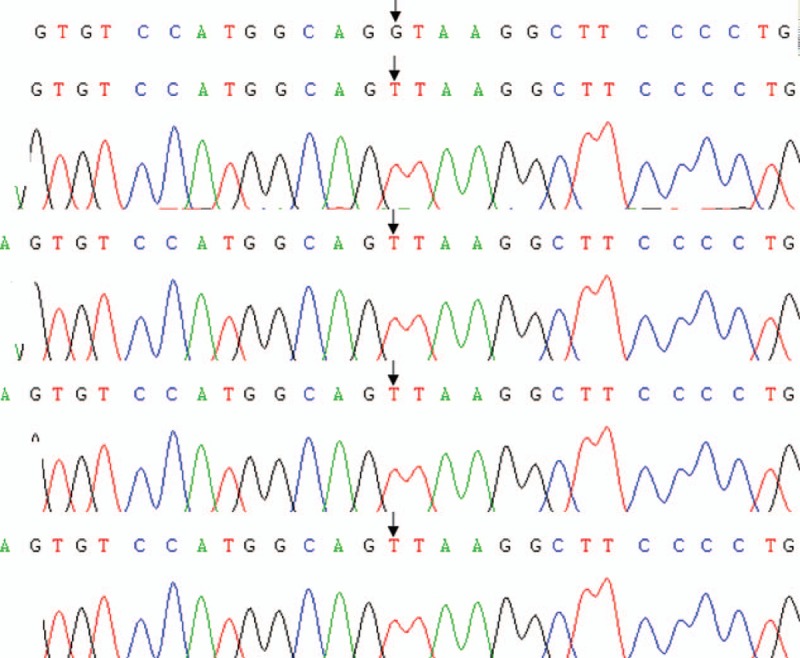
Genetic sequencing results of the patient in case 1, and of her parents and brother. Upon comparison against the NCBI reference sequence that contained anormal variant of the F7 gene (IVS7+1G), the mutation IVS7+1G>T was recognized in the patient, her parents, and her brother. The patient was diagnosed as having homozygous mutation, whereas the parents and brother were identified as heterozygous carriers.

**Figure 4:**
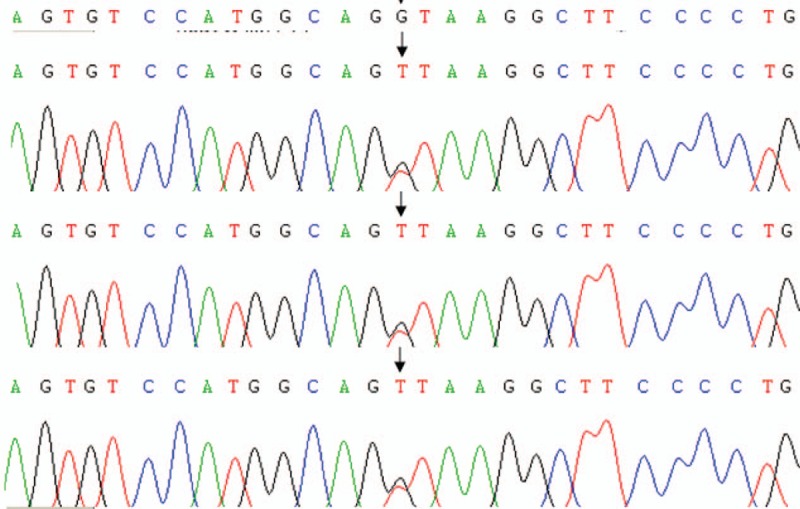
Genetic sequencing results of the patient in case 2 and of her parents. Upon comparison against the NCBI reference sequence that contained a normal variant of the F7 gene (IVS7+1G), the mutation IVS7+1G>T was recognized in the patient and her parents. The patient was diagnosed as having homozygous mutation, whereas her parents were identified as heterozygous carriers.

Genetic analysis was performed to clarify the mutational status of F7 gene, which lies on chromosome 13 and encodes for FVII. The 2 children with FVIID were considered probands, and the genetic study included the 2 patients, their parents, and the brother of the first patient. Genetic testing was conducted by the Beijing Deyi Oriental Translational Medicine Research Center, China. The homozygous splice site mutation IVS7+1G>T was noted in both probands. The mutation was located at the classical cleavage site; this site is not reported before. The tested family members were identified as heterozygous carriers of the IVS7+1G>T mutation (Figs. 3 and 4).

## 3 Literature review

To investigate the clinical features, diagnosis, and treatment of FVIID in newborns, we conducted a thorough review of relevant literature on FVIID. CNKI, WanFang, and PubMed databases were searched for relevant articles published from 1966 to 2017 using the keywords of “congenital; hereditary; inherited; neonate; neonatal; newborn; intracranial hemorrhage; factor VII deficiency” in Chinese and English, respectively. There were 5 Chinese articles^[7–11]^ and 9 English articles.^[12–20]^ concerning FVIID in the neonatal period. Complete clinical data from 19 cases of FVIID in the neonatal period were analyzed, including 10 males (52.6%) and 9 females (47.7%), all of which were full-term neonates. Consanguineous marriage was in 3 cases (15.8%), and positive family history or compatriots with FVIID in 4 cases (21.1%). Onset time ≤7 days was in 14 cases (73.7%), and onset time >7 days was in 5 cases (26.3%). The first symptoms were gastrointestinal bleeding in 6 cases (31.6%), neurological symptoms (drowsiness, convulsions, poor response, among othrers) in 5 cases (26.3%), pallor in 6 cases (31.6%), and bleeding in other sites in 2 cases (10.5%). Intracerebral hemorrhage was observed in 16 cases (84.2%), and gastrointestinal hemorrhage in 7 cases (36.8%). Auxiliary examinations showed normal APTT and prolonged PT (24.6–141 seconds; 20–45 seconds in 8 cases, 45–60 seconds in 3 cases, >60 seconds in 4 cases). FVII activity ranged from 0.9% to 24.6%, and FVII <5% was in 17 cases (89.5%). Nine cases (47.4%) were genetically diagnosed with FVIID. Sixteen cases (84.2%) were treated with fresh frozen plasma, 5 cases (26.3%) underwent prothrombin complex concentrates, and 2 cases (10.5%) received recombinant FVII. Death and severe sequelae occurred in 13 cases (68.4%; Table 2)

**Table 2:**
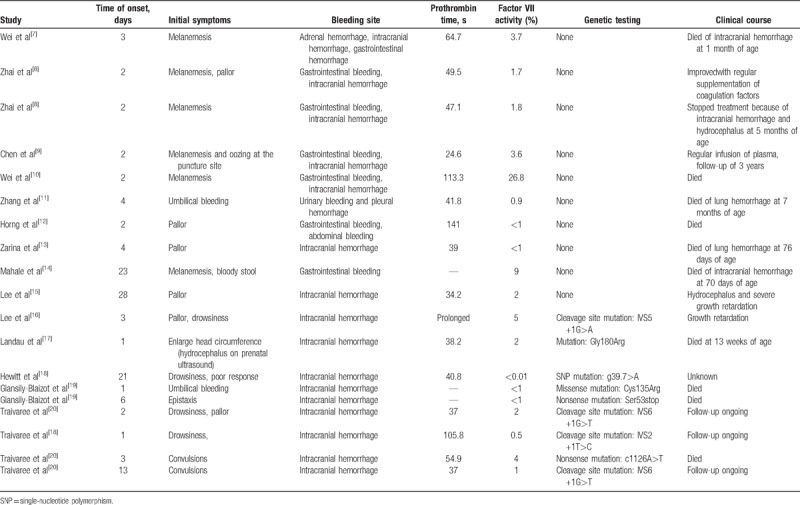
Clinical data of previously described patients with factor VII deficiency in the neonatal period.

## 4 Discussion

FVIID was first reported by Alexander et al in 1951.^[1]^ In China, FVIID was first reported in 2000.^[21]^ FVIID is a rare hereditary bleeding disorder with an incidence of approximately 1/500000.^[3]^ In recent years, the reported incidence rate has risen to 1/26000 (http://www.iss.it/binary/publ/cont/12_55_web.pdf). Both sexes can develop FVIID, and about 18% of patients have a family history of consanguineous marriage. Both neonates described in our report were female and had no family history of consanguineous marriage. Among the previously reported cases of neonatal FVIID, the proportion of males and females were similar, and a family history of FVIID history in 21.1% of cases.

FVII is a vitamin K-dependent glycoprotein synthesized and secreted by the liver. FVII contributes to the initiation of the exogenous coagulation pathway. The activity of FVII in normal blood circulation is 70% to 120%. One-third of neonates with FVIID are diagnosed late and receive delayed treatment.^[22,23]^ Clinically, neonates with FVIID often present with hemorrhagic manifestations as well as unspecific manifestations such as vomiting, fever, pallor, and feeding difficulties. In our patients, physical examinations revealed pallor or skin ecchymosis. Based on our experience and the reports published to date, neonatal FVIID is mainly characterized by normal APTT and prolonged PT that does not respond to multiple doses of vitamin K1 (non–vitamin K-dependent coagulopathy), in combination with very low FVII activity (<5%). Therefore, FVIID should be considered if neonatal patients present with clinical prolongation of PT and non–vitamin K-dependent coagulation disorder and should be checked using further coagulation factor tests.

The clinical manifestations of FVIID vary substantially. Mild cases generally present with minor or post-traumatic hemorrhages such as epistaxis, skin and mucosal petechiae, gingival bleeding, menorrhagia, and persistent bleeding after trauma, whereas severe cases present with life-threatening hemorrhages such as intracranial hemorrhage, gastrointestinal bleeding, and joint bleeding. Severe manifestations occur in 4.4% to 8% of patients with FVIID.^[15]^ Alam et al^[24]^ reported that adult FVIID is mostly characterized by nonfatal bleeding such as repeated epistaxis, skin ecchymosis, menorrhagia, and persistent bleeding after trauma or extraction. Timely symptomatic treatment for adult FVIID is only required after surgery or trauma, as adult FVIID patients often present with late onset, mild conditions, and good prognosis. However, early-onset FVIID in newborns and infants carries a high risk of large intracranial hemorrhage, which is associated with high disability and mortality.^[4]^ FVIID in the neonatal period is rare, with only 19 cases reported to date, and mostly characterized by gastrointestinal bleeding or neurological symptoms as initial symptoms, although some patients may present with umbilical hemorrhage, bleeding at the puncture site, or nasal bleeding. Among the 19 cases of neonatal FVIID reported previously, 16 (84.2%) exhibited intracranial hemorrhage, which was sometimes accompanied by adrenal hemorrhage, pulmonary hemorrhage, chest and intraabdominal hemorrhage, or urinary system bleeding; of the 19 patients, 13 (68.4%) died or developed severe sequelae with extremely poor prognosis. The patient described presently as case 1 initially presented with gastrointestinal bleeding at 7 days after birth, and only a small amount of nonfatal subarachnoid hemorrhage was found on imaging; the patient was diagnosed with FVIID and discharged after symptomatic treatment, but the disease relapsed at 3 days after discharge because of the lack of continuous replacement therapy in the local hospital, and the patient eventually died of severe intracranial hemorrhage. The patient presently described as case 2 also presented with gastrointestinal bleeding as the initial symptom,which improved after treatment; however, intracranial hemorrhage recurred several days later, at 2 months, and 6 months of age, eventually resulting in death. Based on our experience with these 2 cases, as well as the literature published to date, it seems that neonatal FVIID may have severe poor prognosis even if clinical improvement is improved after the initial treatment, especially intracranial hemorrhage, had high mortality, different from FVIID in adult.

By February 2014, 283 FVII gene mutations had been published in the Human Gene Mutation Database (http://www.hgmd.cf.ac.uk/ac/all.php), including 180 missense and nonsense mutations, 39 cleavage site mutations, and 33 small insertion or deletion mutations. Patients with homozygous and compound heterozygous mutations generally develop serious clinical bleeding, whereas those with simple heterozygous mutations and moderate FVII activity (16%–32%) may have no clinical manifestations at all. Herrmann et al^[25]^ proposed that the activity of FVII has no significant correlation with clinical manifestations but is related to the type of gene mutation. Some patients with FVII activity <2% may have only mild or even no clinical manifestations, whereas some patients with FVII activity >5% can develop severe bleeding. Therefore, the severity of bleeding mainly depends on the number of mutations in the FVII gene, the influence of the mutation on the function of FVII, as well as on the gene polymorphism.^[9,10,26]^

Among the 9 previously described patients with neonatal FVIID diagnosed genetically, one had a missense mutation, 2 had nonsense mutations, 6 had cleavage site mutations, 1 had a point mutation, and 1 had a single-nucleotide polymorphism. The family members (parents and sibling) of our 2 patients all had heterozygous mutations with no clinical manifestations. Among the previously reported cases of neonatal FVIID, patients with FVII activity of 26.8% died of severe gastrointestinal bleeding and intracranial hemorrhage. Therefore, the relationship between FVII activity and clinical severity of FVIID remains to be clarified. Landau et al^[17]^ reported 2 cases of severe intracranial hemorrhage during the neonatal period and concluded that presence of the Gly180Arg mutation may have altered the function of the FVII protein, leading to hemorrhage. Tamary et al^[27]^ proposed that IVS2+1G>C and Phe24 mutations can cause life-threatening intracerebral hemorrhage and contribute to prenatal diagnosis. Cavallari et al^[28]^ also reported that splice site IVS6+1G>T mutations can cause life-threatening bleeding. Giansily-Blaziot et al^[19]^ concluded that IVS4+1G>A and Cys135Arg mutations may result in severe intracerebral hemorrhage and are helpful for prenatal diagnosis using genetic testing. The 2 cases of neonatal FVIID reported in our study presented with intracranial hemorrhage and gastrointestinal hemorrhage as their initial symptoms and were diagnosed with FVIID based on the presence of vitamin K1-refractory PT prolongation and severely decreased FVII activity. Genetic sequencing results revealed splice site mutations in intron 7 of the FVII gene in both families evaluated. FVII gene mutations at this site have not been reported in any established databases such as the Online Mendelian Inheritance in Man, Human Gene Mutation Database, and CLinvar database. Discovery of this new mutation site indicates that there may be many genetic families of FVIID-related FVII gene mutations. The mutations identified in our neonatal patients are not registered in the relevant databases. It is possible that this mutation was not previously characterized because they are specific to neonatal FVIID, which is rare. Although adult FVIID is characterized by late onset, mild manifestations, and good prognosis, FVIID in the neonatal period is characterized by early onset, severe manifestations, and very poor prognosis. The association between specific genetic mutations and the pathogenesis of coagulation disorders remains to be clarified. Such knowledge is of great importance for the genetic diagnosis of neonatal FVIID, as well as for developing nonreplacement treatments.

Vitamin K supplementation is ineffective in FVIID, and there is currently no radical treatment for FVIID. Therapeutic strategies mainly rely on infusion of fresh frozen plasma, prothrombin complex concentrates, and human recombinant activating FVII, but the outcomes have been inconsistent. As FVII half-life is very short, ranging from 3 to 6 hours, infusions must be administered frequently. Fresh frozen plasma infusion is inexpensive and easily accessible but can result in blood transfusion-related diseases and is not always effective. Infusion of prothrombin complex concentrates increases the levels of other coagulation factors. Finally, infusion of human recombinant activating FVII is widely used because of high effectiveness and low risk. Nevertheless, this method has some limitations including high cost, reduced availability, and lack of well-established recommendations. Some authors suggest that recombinant activating FVII infusion should be repeated at an interval of 4 to 6 hours and a dosage of 15 to 30 μg/kg until the bleeding has stopped, with prolonged treatment duration in patients with severe bleeding.^[15,29]^ Other authors^[30]^ suggest that injection of recombinant FVII at a dosage of 20 to 30 μg/kg 1 to 2 times per week can prevent FVIID-induced bleeding, but this recommendation still remains controversial. Neonatal FVIID often presents with severe intracerebral hemorrhage,^[8]^ which is difficult to manage in the long term. All the 19 previously described patients received fresh frozen plasma and prothrombin complex infusion. Only 1 received FVII therapy after discharge, and the treatment cost was extremely high. FVIID in the neonatal period is often complicated by severe intracranial hemorrhage that results in death. Therefore, investigation of the possible FVII mutations is necessary. Genetic counseling and prenatal diagnosis are particularly important for families with FVII mutations.

## 5 Conclusion

The clinical manifestations of FVIID in neonates are different from FVIID in adults; we insist it is necessary to inform that:^[24]^ there is currently no alternative treatment for FVIID in the neonatal period; there is a high risk of repeated bleeding, especially severe intracerebral hemorrhage, during infancy; and unlike adult FVIID, neonatal FVIID is associated with high mortality and morbidity.

## Author contributions

**Conceptualization:** Xiaowen Chen.

**Formal analysis:** Xiaowen Chen, Ling Wang.

**Project administration:** Wei Zhou.

**Writing – original draft:** Juan He, Li Tao.

**Writing – review & editing:** Juan He, Wei Zhou, Hui Lv.
